# A case report on mixed pulmonary infection of *Nocardia nova, Mycobacterium tuberculosis*, and *Aspergillus fumigatus* based on metagenomic next-generation sequencing

**DOI:** 10.3389/fpubh.2022.927338

**Published:** 2022-09-06

**Authors:** Haiming Yan, Zhandong Li, Han Xia, Qixin Li, Honglian Bai

**Affiliations:** ^1^Department of Infectious Diseases, The First People's Hospital of Foshan, Foshan, China; ^2^Department of Scientific Affairs, Hugobiotech Co., Ltd., Beijing, China; ^3^Department of Laboratory, The First People's Hospital of Foshan, Foshan, China

**Keywords:** *Nocardia nova*, mixed infection, pulmonary infection, metagenomic next-generation sequencing (mNGS), pathogen diagnosis

## Abstract

**Background:**

Pulmonary infection is one of the common complications of long-term use of glucocorticoids. Severe infections not only increase the length of hospital stay and treatment costs but also cause progression or recurrence of the primary disease.

**Case description:**

Herein, we reported a case of mixed pulmonary infection secondary to glucocorticoid use. Rare pathogens such as *Nocardia nova, Mycobacterium tuberculosis, Aspergillus fumigatus*, and cytomegalovirus were detected by metagenomic next-generation sequencing (mNGS) of bronchoalveolar lavage fluid and lung puncture tissue. Combining the results of conventional pathogen detection and clinical symptoms, the patient was diagnosed with mixed pulmonary infection by multiple pathogens. After timely targeted medication, the patient was finally discharged with a good prognosis.

**Conclusion:**

To our knowledge, this is the first case report on mixed pulmonary infection with pathogens including *Nocardia nova, Mycobacterium tuberculosis, Aspergillus fumigatus*, and human cytomegalovirus. As a new clinical diagnostic method, mNGS has great advantages in diagnosis of diseases such as mixed infections.

## Introduction

Glucocorticoids play an important role in treatment of chronic kidney disease and can be used to treat a variety of kidney diseases. However, long-term use of glucocorticoids can bring about a series of adverse effects, of which infectious diseases, especially pulmonary infections, are one of the common complications. Severe infections will not only increase the length of hospital stay and treatment costs of patients but are also risk factors for progression or recurrence of the primary disease (1). Especially in immunosuppressed populations on glucocorticoids, severe infections often lead to poor prognosis. Rapid and accurate diagnostic tools are urgently needed.

*Nocardia nova* is a Gram-positive aerobic bacterium with weak positive acid-fast staining and is isolated from soil. It has been reported to cause primary skin diseases in immunocompetent individuals, primary sternal osteomyelitis, and brain abscesses in immunosuppressed patients (2), but no pulmonary infections with *N. nova* complicated by tuberculosis or fungi have been reported. A case of secondary mixed pulmonary infection with *N. nova, Mycobacterium tuberculosis, Aspergillus fumigatus*, cytomegalovirus, and other pathogens after glucocorticoid use in our hospital is reported. mNGS is finally applied, and the pathogens are successfully diagnosed in this patient.

## Case presentation

A 57-year-old man was admitted because of abdominal distension, anorexia and constipation for 5 weeks, chest pain for 1 week, and fever for 1 day on 16 April 2020. On 8 March 2020, the patient developed abdominal distension, anorexia, constipation, and decreased anus exhaust, and was given drugs to promote gastrointestinal motility but with no improvement. On 9 April 2020, the patient was accidentally hit by a wrench on the left side of the chest at work, and it was accompanied by chest pain. The chest CT at the local hospital revealed Wegener's granulomatosis, which did not exclude the possibility of tuberculosis, and no further diagnosis or treatment were performed. On 15 April 2020, the patient developed chills and fever with a body temperature of 39C during his visit to our outpatient department. The blood routine showed a WBC of 7.33 × 10^9^/L, hemoglobin of 98 g/L, a neutrophil ratio of 87.1%, and a C-reactive protein of 119.72 mg/L. The results of plain chest and abdomen films showed multiple lesions in bilateral lungs, with the nature to be determined, and enterostasis. Then, the patient was admitted for further treatment.

The patient had chronic viral hepatitis B and type 2 diabetes mellitus, and was given entecavir 0.5 mg qd for anti-hepatitis B virus treatment. He was diagnosed with hepatitis B virus-associated membranous nephropathy in 2019 and had received high-dose hormone therapy (Medrol 48 mg qd) for 2 months before the onset of the disease. His lymphocytes were decreased: total T lymphocytes 427 cells/μl, helper T cells 210 cells/μl, and suppressor T cells 209 cells/μl. Two months ago, the patient was given methylprednisolone 48 mg qd because there was no improvement in urine protein, and the dose was reduced to 44 mg qd 2 days before the admission. The patient was engaged in furniture manufacturing and works in a dusty environment.

On admission, his body temperature was 39.5°C, the respiratory rate was 22 breaths per minute, and his consciousness was clear. There was no yellowing of the whole body skin mucosa, rash, or subcutaneous hemorrhage. Liver palm was notable but without obvious spider nevus. The oral cavity and pharynx were found to have a bean curd-like attachment, and the tongue was thick with white spots. The patient's breath sounds were weak bilaterally, with the left lung more pronounced, and there was pressure pain around the umbilicus. The cardiovascular system and nervous systems and the limbs were unremarkable.

The laboratory tests showed interleukin-6 of 137.2 pg/ml, serum ferritin of 1,642.3 ng/ml, and an erythrocyte sedimentation rate of 83 mm/h. The T-lymphocyte subsets shown by flow cytometry indicated an absolute value of total T cells of 427 cells/μl, an absolute value of helper T cells (Th) of 210 cells/μl, and an absolute value of suppressor T cells (Ts) of 209 cells/μl. Seven items of immune functions demonstrated a total complement hemolytic activity of 68 IU/L and immunoglobulin G of 6.29 g/L. For liver function, globulin was 19.3 g/L and albumin was 24.6 g/L. Glycosylated hemoglobin was 10.7%. The tuberculosis immune spot test was negative. Fungal D-glucan was 745 ng/L. The fungal culture with throat wipes detected *Candida albicans*. The routine urine test was positive for urine protein and urine glucose.

After the admission, the patient was given fluconazole injection 200 mg qd, latamoxef 1 g q12 h for anti-infection, entecavir 0.5 mg qd for anti-hepatitis B virus, methylprednisolone 44 mg qd for anti-hepatitis B virus, and blood glucose control, urine protein reduction, kidney protection, and other treatments. On the 2nd day of admission, the patient's temperature returned to normal, and the re-examination showed a high-sensitivity C-reactive protein of 20.98 mg/L and procalcitonin <0.05 ng/ml. Since the pathogens of pulmonary infection were unknown, on the 7th day of admission, bronchofiberscopy was performed and bronchoalveolar lavage fluid (BALF) was collected for pathogen detection (culture + tuberculosis gene probe). The patient presented with a postoperative transient temperature of 38.5°C with chills and was considered to have an acute upper respiratory tract infection. After antiviral treatment with oseltamivir 75 mg bid, the body temperature returned to normal on the same day. Sputum specimens and BALF specimens were sent several times for sputum smears and cultures, and no *M. tuberculosis* was found in the smears or cultures. However, the BALF specimens were positive for *M. tuberculosis* gene probes A, B, C, and E and negative for rifampicin resistance mutation. *A. fumigatus* was cultured from the bronchoalveolar lavage fluid, and the preliminary diagnosis was pulmonary tuberculosis and pulmonary *Aspergillus* infection. Glucocorticoids were discontinued on the 8th day of admission, and fluconazole and latamoxef were discontinued. The anti-tuberculosis regimen was changed to isoniazid 0.3 g qd, ethambutol 1 g qd, pyrazinamide 1 g qd, and levofloxacin 0.4 g qd, and the antifungal therapy was changed to Voriconazole 200 mg q12 h.

**Figure 1:**
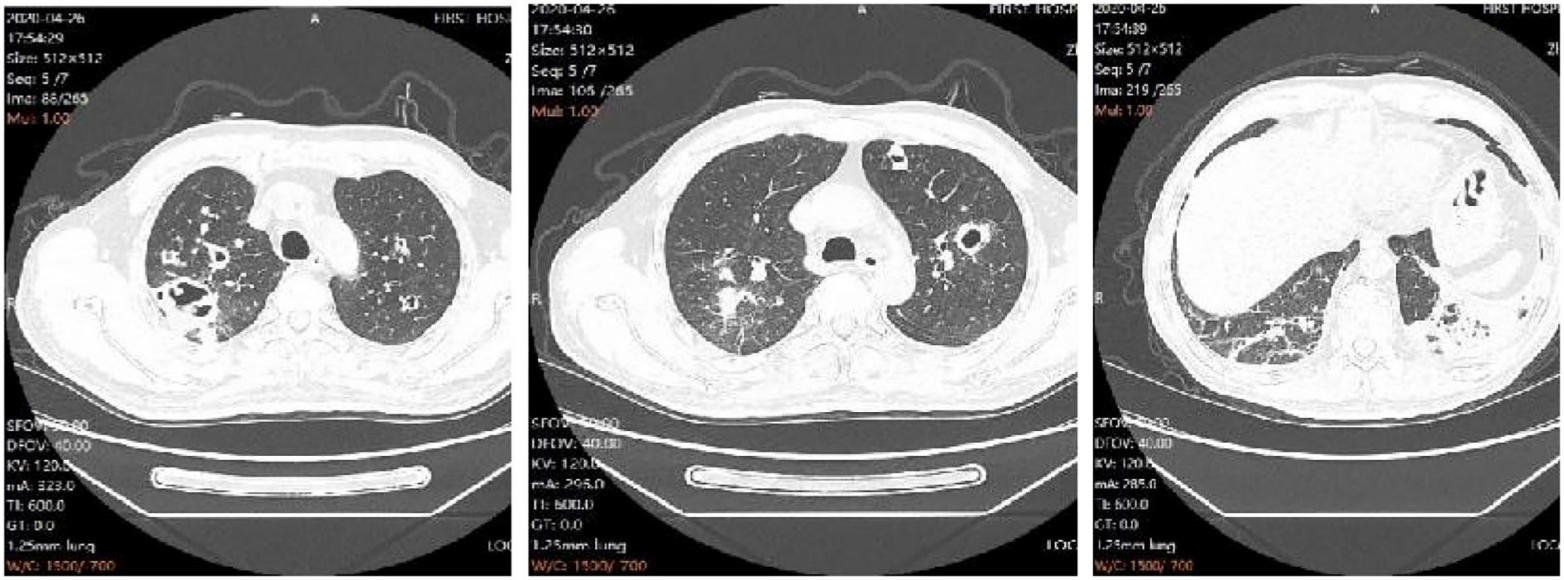
Radiographic progression of lungs.

On the 10th day of admission, the patient again developed hyperthermia, with a temperature of 39.4°C, accompanied by chills, left-sided chest pain with blood in the sputum, and shortness of breath. The repeat examination showed high-sensitivity a C-reactive protein of 98.58 mg/L, procalcitonin of 0.09 ng/ml, and a decreased oxygenation index compared to before. The repeat chest CT showed multiple infectious lesions, some nodules with cavities in both upper lungs, a small amount of pleural and pericardial effusion, and progressive lesions compared with the CT film from another hospital (Figure 1). Considering that the fever was caused by aggravation of the pulmonary infection, levofloxacin was changed to moxifloxacin hydrochloride in sodium chloride injection 0.4 g qd. Fever peak decreased slightly, but the body temperature did not decrease to normal. On the 13th day of admission, the patient had a high fever again, with the highest body temperature of 39.5°C. The infection indicators continued to rise on recheck, with a high-sensitivity C-reactive protein of 123.54 mg/L and procalcitonin of 0.11 ng/ml.

**Figure 2:**
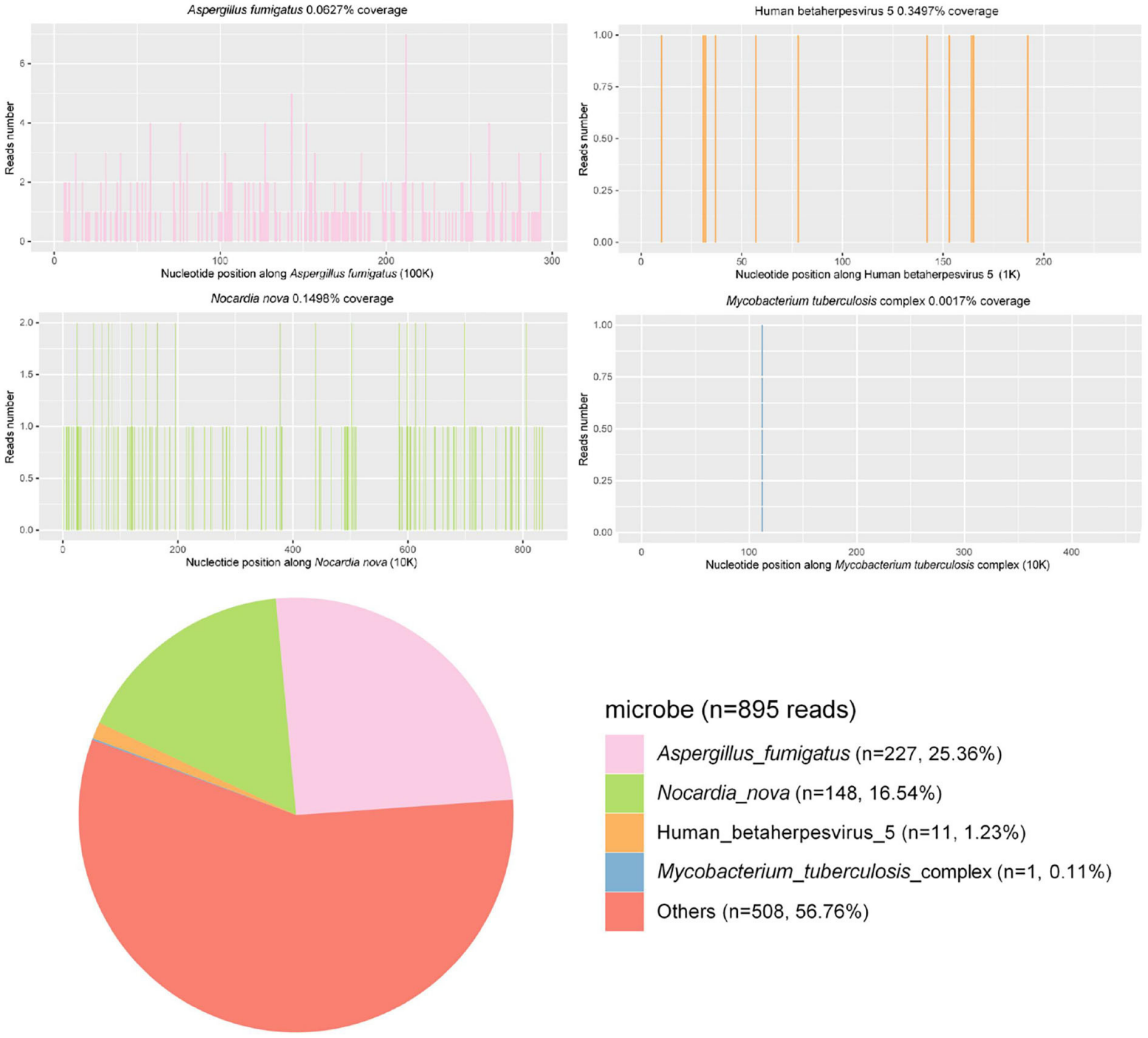
Results of the metagenomic next-generation sequencing.

BALF samples were submitted for PACEseq metagenomic next-generation sequencing (mNGS) (Hugobiotech, Beijing, China), and *A. fumigatus* (227 reads), *N. nova* (148 reads), *M. tuberculosis* (1 read), human alphaherpesvirus 1 (39 reads), and human cytomegalovirus (11 reads) were detected (Figure 2). Briefly, for the mNGS method, the collected BALF specimen was centrifuged at 5,000 g and at room temperature for 10 min, and DNA was extracted from the supernatant using the TIANamp Micro DNA Kit (DP316, Tiangen Biotech). Sequencing libraries were constructed with the QIAseq ™ Ultralow Input Library Kit (Illumina). Qualified libraries with different tags were pooled together and amplified and then sequenced with a Nextseq550 system (Illumina) for 150 cycles. *Kytococcus sedentarius* (51 reads), *Streptococcus gordonii* (36 reads), *Rothia dentocariosa* (82 reads), *Nocardia mikamii* (10 reads), *Streptococcus mitis* (21 reads), *Lactobacillus salivarius* (25 reads), and *Streptococcus pseudopneumoniae* (43 reads) were also detected by the mNGS but were considered as oral colonizing microorganisms and were not considered as pathogenic microorganisms.

**Figure 3:**
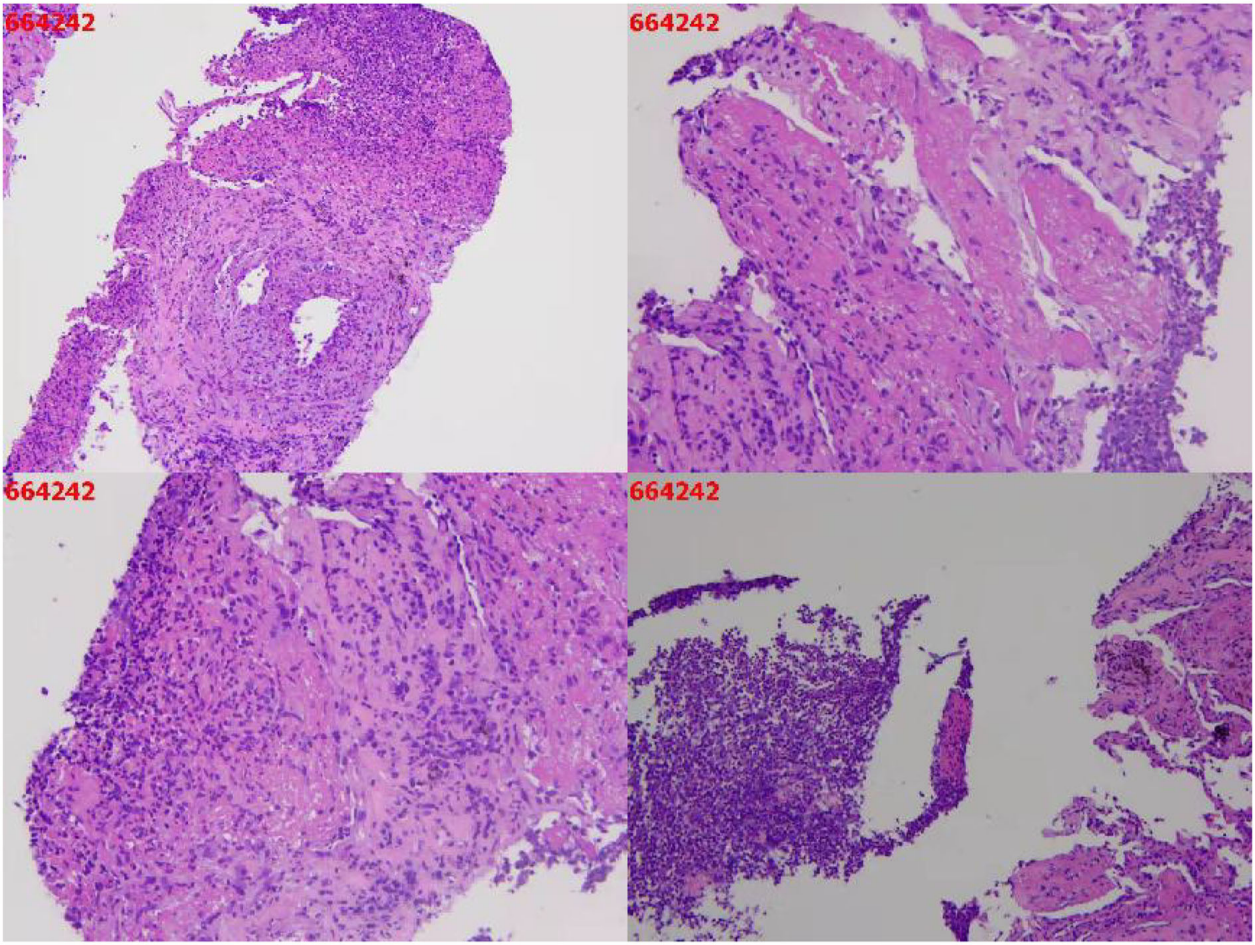
Histopathological results of the lungs.

A needle biopsy of the left upper lung tissue was also performed, and the mNGS of the lung tissue revealed *N. nova* (92 reads) and human cytomegalovirus (2 reads) (Figure 2). The serum viral examination was CMV-IgG positive and CMV DNA-positive. Herpes simplex virus type I IgG was positive and herpes simplex virus type I IgM was negative. The histopathological examination of the lungs revealed purulent inflammation with fibrinous inflammation (Figure 3). Combined with the characteristics of imaging changes, mNGS results of BALF and lung tissues, the patient was considered to have a multi-pathogen infection (*N. nova, M. tuberculosis, A. fumigatus*, and cytomegalovirus). The pulmonary imaging features of this patient were predominantly cavernous and not consistent with viral pneumonia manifestation features, so respiratory infection caused by human alphaherpesvirus 1 was not considered.

**Figure 4:**
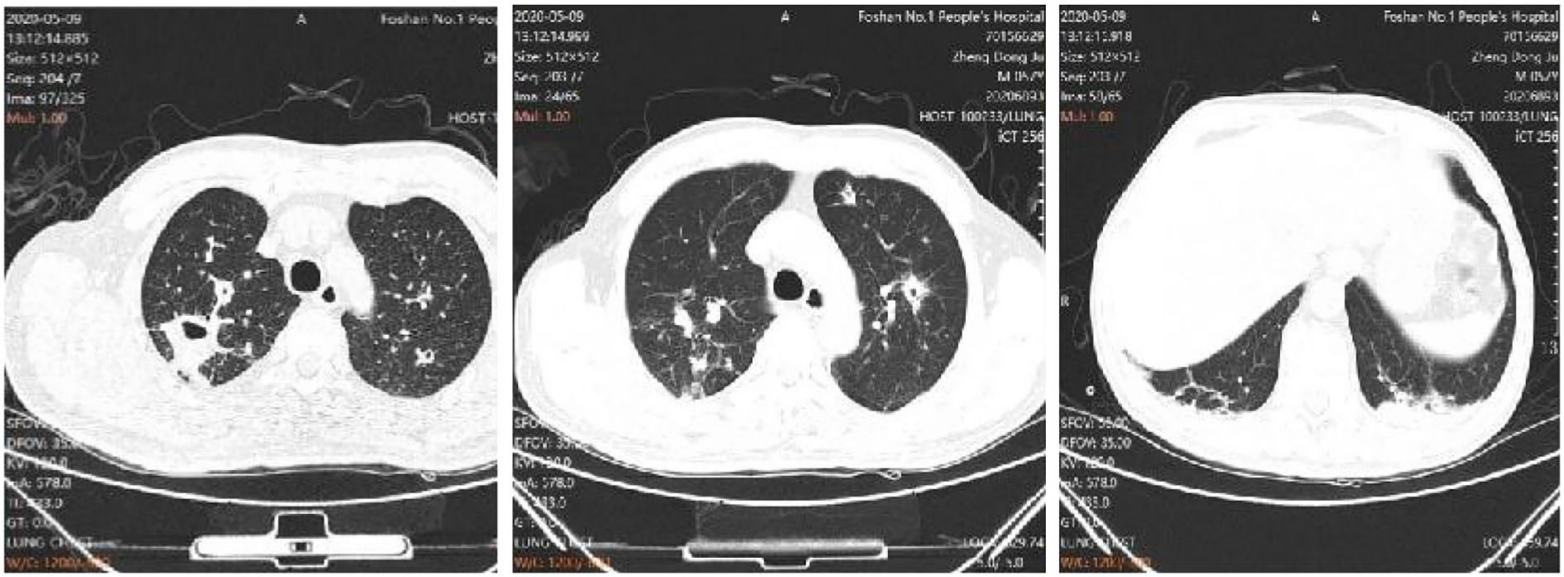
Imaging of the lungs after treatment.

The treatment regimen was then adjusted to co-trimoxazole 3 tablets tid and imipenem and cilastatin sodium injection 1 g, q8 h for the treatment of *N. nova*, and ganciclovir 0.25 g, q12 h for the treatment of cytomegalovirus. The anti-tuberculosis and *Aspergillus* treatments were also continued. The fever peak was decreased, and his chest pain was relieved, with normal temperature on the 17th day of admission. The dynamic reexamination revealed that the infection indicators had decreased gradually. On day 24 of admission, the repeat chest CT showed improvement of the pulmonary infection (Figure 4), CMV DNA was negative, and “ganciclovir” was discontinued. On day 44 of admission, the patient recovered and was discharged. The patient continued to receive the anti-tuberculosis and antifungal treatments with Voriconazole 200 mg q12 h and sulfonamide Tablets 3 tablets tid for *N. nova* in the outpatient department. A review of the chest CT at 1 and 3 months after discharge showed that the lesion was gradually absorbed.

## Discussion

In this case, the patient had positive results for the *M. tuberculosis* gene probe test and fungal culture for *A. fumigatus*, and was initially diagnosed with tuberculosis combined with pulmonary *Aspergillus* infection. However, his condition did not improve after the anti-tuberculosis and anti-fungal treatments. At this point, mNGS revealed that the pathogens involved not only *M. tuberculosis* and *A. fumigatus* but also CMV and the rare pathogen *N. Nova*. The mNGS of lung tissues showed that *N. nova* infection was indeed present. After the addition of Co-trimoxazole, imipenem and cilastatin sodium injection for the treatment of *N. nova*, and ganciclovir for the treatment of cytomegalovirus, the patient's symptoms were relieved.

*Nocardia* is a Gram-positive rod-shaped aerobic mycobacterium belonging to the order Actinomycetales; It has weak acid tolerance and is widely present in soil, air, dust, freshwater, seawater, and decaying plants (3). This bacterium is not a normal parasitic bacterium of the human body and occurs mostly in people with T-cell immunodeficiency, such as those with AIDS and hematological diseases, after hematopoietic stem cell transplantation, solid organ transplantation, with connective tissue diseases, tumor radiotherapy and chemotherapy, and long-term use of hormones and/or immunosuppressive agents (4, 5). *A. fumigatus* and *N. nova* have similar susceptibility factors, and the most frequent organ invaded by both is the lungs. Tuberculosis infections can occur in immunocompetent individuals, and are more common in immunocompromised patients. All the three pathogens have similar clinical symptoms including fever, cough, sputum with blood streaks, chest pain, chest tightness, shortness of breath, weight loss, fatigue, and night sweats, so they are often misdiagnosed and mistreated in clinical practice, and a definitive diagnosis must rely on etiological detection results.

Clinical diagnostic methods, including serology and culture, usually target common pathogens with low sensitivity. For the culture of rare pathogens, fastidious pathogens, viruses, and mixed infections, traditional methods often have limitations. As an emerging pathogen diagnostic method, mNGS has excelled in the clinical diagnosis of a variety of infectious diseases. It has an ability to detect pathogens in samples without bias and has a great advantage for diagnosis of mixed infections.

Many co-infection cases of *Nocardia* and pulmonary *Aspergillus* are reported in the literature. Jianyong Wu et al. reported a case of pulmonary infection caused by Nocardia combined with *A. fumigatus* in a 55-year-old woman, and the *Nocardia* was identified by MALDI-TOF MS and 16S rRNA gene sequencing, indicating that early and accurate diagnosis and treatment are essential for severe pulmonary infection with *Nocardia* (6). Meena et al. reported a case of pulmonary tuberculosis complicated by Nocardia infection in a 46-year-old woman with COPD. This infection can be easily misdiagnosed because the clinical features are non-specific, and all pathogens are difficult to culture (7). However, there is no relevant literature report on mixed pulmonary infection of *Nocardia, M. tuberculosis*, Aspergillus, and cytomegalovirus infection in China and abroad. In this case, only *M. tuberculosis* and *A. fumigatus* were detected with the conventional method, and the patient's condition did not return to normal after medication. Then, by mNGS, *N. nova* and cytomegalovirus were detected in addition to *M. tuberculosis* and *A. fumigatus*, indicating that mNGS is highly advantageous in diagnosing rare pathogens, viruses, and pathogens of mixed infections.

This is the first case of mixed infection of the four pathogens confirmed by mNGS technology, and after timely anti-infective treatment, the patient recovered and was discharged. Meanwhile, this case suggests that mNGS has outstanding advantages and value in detection of pathogens of rare and mixed pulmonary infections (8), and can further help clinicians with rare mixed infections and prevent misdiagnosis and underdiagnosis.

## Data availability statement

The datasets presented in this study can be found in the National Genomics Data Center (https://ngdc.cncb.ac.cn/), accession no. PRJCA008186.

## Author contributions

HY, QL, and HB designed and drafted the article. ZL was involved in the clinical care and management of the patient. HX analyzed the mNGS data. All authors approved the final version of the manuscript as submitted and agreed to be accountable for all aspects of the study.

## Funding

The research was supported by the Science and Technology Project of Xi'an (No. 21RGSF0013).

## Conflict of interest

Author HX was employed by Hugobiotech Co., Ltd. The remaining authors declare that the research was conducted in the absence of any commercial or financial relationships that could be construed as a potential conflict of interest.

## Publisher's note

All claims expressed in this article are solely those of the authors and do not necessarily represent those of their affiliated organizations, or those of the publisher, the editors and the reviewers. Any product that may be evaluated in this article, or claim that may be made by its manufacturer, is not guaranteed or endorsed by the publisher.
